# Identification and Characterization of a Novel Association between Dietary Potassium and Risk of Crohn’s Disease and Ulcerative Colitis

**DOI:** 10.3389/fimmu.2016.00554

**Published:** 2016-12-07

**Authors:** Hamed Khalili, Sakshi Malik, Ashwin N. Ananthakrishnan, John J. Garber, Leslie M. Higuchi, Amit Joshi, Joanna Peloquin, James M. Richter, Kathleen O. Stewart, Gary C. Curhan, Amit Awasthi, Vijay Yajnik, Andrew T. Chan

**Affiliations:** ^1^Division of Gastroenterology, Massachusetts General Hospital, Harvard Medical School, Boston, MA, USA; ^2^Clinical and Translational Epidemiology Unit, Massachusetts General Hospital, Boston, MA, USA; ^3^Translational Health Science and Technology Institute, NCR Biotech Science Cluster, Faridabad, India; ^4^Division of Gastroenterology and Nutrition, Boston Children’s Hospital, Harvard Medical School, Boston, MA, USA; ^5^Division of Gastroenterology and Hepatology, Johns Hopkins School of Medicine, Baltimore, MD, USA; ^6^Channing Division of Network Medicine, Brigham and Women’s Hospital, Harvard Medical School, Boston, MA, USA

**Keywords:** inflammatory bowel disease, Crohn’s disease, ulcerative colitis, dietary potassium, immune tolerance, T_H_17 pathway, Nurses’ Health Study

## Abstract

**Background:**

Recent animal studies have identified that dietary salt intake may modify the risk and progression of autoimmune disorders through modulation of the IL-23/T_H_17 pathway, which is critical in the pathogenesis of ulcerative colitis (UC) and Crohn’s disease (CD).

**Methods:**

We conducted a prospective study of U.S. women enrolled in the Nurses’ Health Study (NHS) and NHSII who provided detailed and validated information on diet and lifestyle beginning in 1984 in NHS and 1991 in NHSII. We confirmed incident cases of UC and CD reported through 2010 in NHS and 2011 in NHSII. We used Cox proportional hazards models to calculate hazard ratios and 95% confidence intervals. In a case–control study nested within these cohorts, we evaluated the interaction between single nucleotide polymorphisms (SNPs) in genes involved in T_H_17 pathway and dietary potassium on risk of CD and UC. In a cohort of healthy volunteers, we also assessed the effect of supplemental potassium on development of naïve and memory T cells, differentiated with TGFβ1 or T_H_17 conditions.

**Results:**

Among a total of 194,711 women over a follow-up of 3,220,247 person-years, we documented 273 cases of CD and 335 cases of UC. Dietary intake of potassium (*P*_trend_ = 0.005) but not sodium (*P*_trend_ = 0.44) was inversely associated with risk of CD. Although, both dietary potassium and sodium were not significantly associated with risk of UC, there was a suggestion of an inverse association with dietary potassium (*P*_trend_ = 0.08). The association of potassium with risk of CD and UC appeared to be modified by loci involved in the T_H_17 pathway that have previously been associated with susceptibility to CD, particularly SNP rs7657746 (*IL21*) (*P*_interaction_ = 0.004 and 0.01, respectively). *In vitro*, potassium enhanced the expression of Foxp3 in both naïve and memory CD4+ T cells *via* activating Smad2/3 and inhibiting Smad7 in T_H_17 cells.

**Conclusion:**

Dietary potassium is inversely associated with risk of CD with both *in vitro* and gene–environment interaction data suggesting a potential role for potassium in regulating immune tolerance through its effect on Tregs and T_H_17 pathway.

## Introduction

Inflammation bowel disease (IBD), comprised of Crohn’s disease (CD) and ulcerative colitis (UC), is a chronic inflammatory disorder of the gastrointestinal tract. Through identification of over 160 risk loci (1–3), genome-wide association (GWA) studies have identified a number of key pathways involved in the development of CD and UC, including the IL-23/T_H_17 axis, autophagy, and mucosal barrier function. However, it is estimated that the risk attributable to genetic predisposition alone is less than 25% (4), highlighting the importance of identifying environmental factors associated with IBD susceptibility. In addition, understanding the interrelationship between genetic markers and environmental factors, also known as gene–environment interaction, can provide insight into the etiopathogenesis of IBD.

Recently, animal studies have shown a role for dietary sodium in the development and progression of autoimmune disorders through inducing IL-23-IL-23R-mediated induction of pathogenic T_H_17 cells, which plays a critical role in development of IBD (5–7). In addition, sodium appears to not only enhance the induction of T_H_17 cells but also inhibit the suppressive functions of Foxp3^+^ Treg cells (8). Sodium may also lead to macrophage activation creating a more inflammatory environment (9). High salt levels inhibit alternative activation of macrophages (M2) resulting in attenuation of tissue inflammation (10). Similarly, sodium impairs Treg function by inducing IFNγ production in these cells. Taken together, these data point to a high sodium diet leading to an enhanced proinflammatory response through interference with both innate and adaptive regulatory mechanisms.

To date, there are limited data on the direct effect of potassium on effector and regulatory T cells. Nevertheless, human and animal data have also supported a link between potassium channels expressed in the intestinal epithelial cells and risk of development of CD or intestinal inflammation in animal models of colitis (11, 12). We therefore sought to prospectively examine the association between dietary intake of sodium and potassium and risk of incident CD and UC within the large, well-characterized Nurses’ Health Study (NHS) and NHSII cohorts. In addition, we aimed to better characterize these associations by examining gene–environment interactions using susceptibility loci previously identified through GWA studies with risk of CD and UC. Finally, we confirmed the functional significance of our findings through *in vitro* studies examining the effect of these minerals on regulatory and effector T cells.

## Materials and Methods

### Cohort Study: Dietary Sodium and Potassium and Risk of UC and CD

#### Study Population

The NHS is a prospective cohort study comprised of nearly 121,700 U.S. women, ages 30–55 years, who completed a mailed health questionnaire starting in 1976. Information on health information has since been updated every 2 years through follow-up questionnaires. NHSII is a similar cohort of 116,686 U.S. women between the ages of 25–42 years that was started in 1989. These women have been followed with similar biennial questionnaires. The Human Research Committee at Partners Healthcare approved this study.

#### Assessment of Diet

Dietary assessment was performed using a 161-item semi-quantitative food frequency questionnaire (SFFQ) starting in 1984 in NHS and updated in 1986 and every 4 years since. Similarly in NHSII, dietary intake was assessed using SFFQ staring in 1991 and updated every 4 years. We calculated nutrient intake from the number of servings of food items and U.S. Department of Agriculture data on the content of nutrients in specified portions. The reproducibility and validity (against dietary records) of the FFQs have been extensively documented (13, 14). All nutrient values were adjusted for total caloric intake by the residual method to estimate intakes independent of the amount of total intake. The correlation between energy-adjusted potassium obtained from FFQ and diet records was 0.53 (15). In addition, the correlation between dietary potassium obtained from FFQ and urinary secretion of potassium was 0.56 in men and 0.39 in women and that of sodium was 0.66 in men and 0.56 in women (16). For questionnaire cycles with no assessment of dietary intake, we carried forward data from the prior questionnaire cycle when this detailed assessment was done. However, we did not carry forward dietary information. Specifically, women with missing dietary data in a 2-year cycle when dietary data was specifically queried did not contribute person-year to the analyses.

#### Assessment of Other Covariates

Detailed information on lifestyle factors, including body weight, smoking status, use of non-steroidal anti-inflammatory drugs (NSAIDs), menopausal hormone therapy, and oral contraceptives were collected and updated in each questionnaire cycle. Information on physical activity was also estimated using an instrument administered every 2–4 years that has been described in detail (17). Participants’ self-report of body weight, height, physical activity, and use of oral contraceptives have been previously validated (18–20). In 1992, in NHS and 1991 in NHSII, women were also asked about their latitude of residence at age 30, which we have previously shown to be associated with risk of UC and CD (21). Information about history of appendectomy was collected in 1992 in NHS and 1995 in NHSII.

Information on ancestry was collected in the 1992 questionnaire in NHS and baseline questionnaire in NHSII. Specifically, women were asked whether they had the following ancestries: African, Asian, Hispanic, Scandinavian, southern European/Mediterranean, other white, or other ancestry. Most of women (93% in the NHS I, 91% in the NHSII) reported white ancestries, consistent with underrepresentation of minorities in healthcare at the inception of both cohorts. More than 90% of the women reported a single ancestry. Ancestry data were available in more than 95% of women in NHS and 99% of women in NHSII.

#### Outcome Ascertainment

Detailed information on confirmation of self-reported cases of CD and UC has been previously reported (17, 21, 22). Briefly, since 1976, participants have reported diagnoses of UC or CD through an open-ended response on biennial surveys. Diagnoses of UC and CD have also been specifically queried since 1982 and 1992, respectively. Similarly in NHSII, both diagnoses were specifically queried starting in 1993. Participants who reported diagnosis of CD or UC on any biennial questionnaire received a supplementary questionnaire. In addition, medical records from these participants were requested and reviewed by two gastroenterologists blinded to exposure information. We used standardized criteria to confirm cases of CD and UC (23–26).

#### Statistical Analysis

For analysis of dietary sodium, potassium, and food items, person-time for each participant was calculated from the date of return of their baseline questionnaires to the date of the diagnosis of UC or CD, date of last returned questionnaire, or June 1, 2010 for NHS and June 1, 2011 for NHSII, whichever came first. At baseline, we excluded participants with missing dietary data and history of IBD or cancer (with the exception of non-melanoma skin). We calculated adjusted hazard ratios (HRs) and 95% confidence intervals (CIs) using Cox proportional hazards modeling incorporating time-varying covariates. Consistent with prior analyses, information on BMI was derived from baseline questionnaire to avoid potential bias from changes in weight by preclinical disease (27, 28). Dietary sodium and potassium were modeled as quintiles while food items were modeled based on number of servings consistent with prior dietary analysis (28, 29). We observed no heterogeneity in the association of dietary sodium, potassium, and food items with CD or UC in separate analyses of NHS and NHSII (*P* for heterogeneity > 0.50 for both UC and CD). Thus, we pooled individual-level data from NHS and NHSII and adjusted for cohort in all analyses. We used the first quintile as the reference category and calculated the risk of CD and UC for each quintile of dietary sodium and potassium by comparing higher quintiles to the first quintile. Using previously described methods, we performed mediation analysis to determine whether the reported association of fiber intake on risk of CD was explained by intake of potassium (30). We used SAS version 9.3 (Cary, NC, USA) for all analyses.

### Nested Case–Control Study: Dietary Potassium, T_H_17 Pathway and Risk of CD and UC

#### Study Population

In 1989–1990, 32,826 NHS participants (aged 43–69 years) returned a blood sample on ice packs by overnight courier and completed a short questionnaire (31). Between 1996 and 1999, 29,611 NHSII participants (aged 32–54 years) provided blood samples and completed a short questionnaire in a similar protocol (32). Similarly in 2001–2004, 29,684 participants in NHS and 29,859 participants in NHSII mailed in a sample of buccal cells collected using a “swish-and-spit” method. Participants who provided the buccal cells had not previously provided a blood specimen. Among participants who provided a blood or saliva specimen, we matched 169 CD cases to 740 controls and 202 UC cases to 740 controls based on age, menopausal status, month of blood collection, and fasting status. Genomic DNA was isolated from buccal cells or blood samples using conventional methods (33).

#### Genotyping and Computation of Risk Score

We used the most recent meta-analysis of GWA studies to identify six single nucleotide polymorphisms (SNPs) in genes involved in T_H_17 pathway that have previously been associated with risk of CD and UC: rs10758669 (*JAK2*), rs12942547 (*STAT3*), rs1819333 (*CCR6*), rs7657746 (*IL21*), rs3024505 (*IL10*), and rs11209026 (*IL23R*) (3). We directly genotyped these SNPs by 5′ nuclease assay (TaqMan^®^), using the ABI PRISM 7900HT Sequence Detection System (Applied Biosystems, Foster City, CA, USA). TaqMan^®^ primers and probes were designed using the Primer Express^®^ Oligo Design software v2.0 (ABI PRISM). The status of individual case–control status was not known to laboratory personnel. Genotyping procedures were validated using duplicates from nearly 10% of the samples; concordance was 100% for these samples. Primers, probes, and conditions for the genotyping assay are available upon request. We confirmed that all SNPs were in Hardy–Weinberg equilibrium among the controls using the Chi-square test (all *P* > 0.30).

We examined associations according to each of the T_H_17 pathway SNPs except rs11209026 (*IL23R*) due to low minor allele frequency (~7%). However, we also constructed two summary risk scores (one for each outcome) incorporating all six T_H_17 pathway SNPs associated with CD and UC risk using a previously described weighting method (34, 35). Briefly, each SNP was recoded as 0, 1, or 2 according to the number of alleles associated with increasing risk of CD or UC; each SNP was weighted by its relative effect size (β coefficient) derived from the previously reported meta-analysis data (separate β coefficient for UC and CD) (3). We constructed the genetic risk score using the equation: genetic risk score = (β1 × SNP1 + β2 × SNP2 + … + β*n* × SNP*n*)/(sum of the β coefficients).

#### Statistical Analysis

We used conditional logistic regression for risk of CD or UC with a multiplicative interaction term for dietary potassium and genotype in T_H_17 pathways defined according to the number of risk alleles while adjusting for other potential risk factors (see Assessment of Other Covariates). Since CD and UC are rare outcomes, we used odds ratios as estimates of relative risks. To minimize the potential influence of reverse causality, we analyzed prospectively collected data on diet from the questionnaires administered 4 years prior to diagnosis of CD or UC for cases and their matched controls. Although, there were no significant variations in allele frequency of genotyped risk variants according to European ancestry, we adjusted all models of gene–environment interaction for ancestry (see Assessment of Other Covariates). We used SAS version 9.3 (Cary, NC, USA) for these analyses. All *P*-values were two-sided and <0.05 was considered statistically significant. As the selection of the SNPs was done *a priori* and interaction with only five SNPs was tested, we did not adjust for multiple testing in our analyses.

### Experimental *In Vitro* Studies: Effect of Potassium on T_H_17/Treg Cell Balance

#### *In Vitro* Human T Cell Differentiation and Intracellular Staining

After obtaining consent, we collected 10 cc of peripheral blood from 25 healthy volunteers, ages 18–35 years without history of HIV, tuberculosis, or any autoimmune diseases (e.g., rheumatoid arthritis, systemic lupus, etc.) and who were not taking steroids or other immunosuppressive medications. All experiments were performed in accordance to the approved guidelines of Human Ethics Committee of Translational Health Science and Technology Institute (THSTI), Faridabad, India.

Peripheral blood mononuclear cells (PBMCs) were isolated by Ficoll Paque density gradient centrifugation (GE Healthcare). Naïve CD4^+^ CD45RO^−^CD25^−^ or memory CD4^+^CD45RO^+^ T cells were sorted as previously described (36). Sorted cells were cultured in 96-well U-bottom plates at a concentration of 0.1 × 10^6^ cells/ml in X-VIVO 15 medium (Lonza) supplemented with 100 units/ml Penicillin/Streptomycin, l-glutamine, NEAA, and Sodium Pyruvate. Cells were stimulated with plate bound anti-CD3 (UCHT1; 10 μg/ml), soluble anti-CD28 (28.2; 3.0 μg/ml), and were differentiated either with TGFβ1 (1 ng/ml) or with T_H_17 condition with addition of following cocktails: TGFβ1 (5.0 ng/ml; R&D), IL-6 (25 ng/ml; Peprotech), IL-1β (12.5 ng/ml; Peprotech), IL-21 (20 ng/ml; Peprotech), and IL-23 (20 ng/ml; Peprotech) for 6 days. Cells were supplemented with or without potassium chloride (Sigma Aldrich). On day 6, cells were stained with LIVE/DEAD Fixable Dead Cell Stain Kit (Invitrogen) according to manufacturer’s protocol. Cells were then fixed and permeabilized with BD Cytofix/Cytoperm and stained with anti-human Foxp3-PE antibody (BD Bioscience; 560082). Labeled cells were acquired on BD FACS Verse and analyzed by FlowJo (Treestar, USA). All FACS plots were gated on live cells.

#### CFSE T Cell Proliferation Assay

For proliferation assay, sorted naïve human CD4^+^ T cells (1 × 10^6^/ml) were labeled with 5.0 μM of CFSE in PBS at 37°C for 5 min. Cells were shaken intermittently in order to get homogenously labeled cell population. Cells were thoroughly washed with glutamine rich media (RPMI-1640) to wash off unlabeled dye. Cells were stimulated with plate bound anti-CD3 and soluble anti-CD28 in round bottom 96-well plate at a concentration of 0.1 × 10^6^/ml with and without KCl (40mM). On day 5, cells were washed extensively with 1× PBS and fixed and permeabilized with Foxp3 staining kit (eBiosciences; 00-5523-00) and were labeled with fluorescent-conjugated Foxp3 antibody (Biolegend, 320014) for 30–40 min on ice. Cells were also stained with live/dead marker to exclude dead population during analysis. Prism 5.0 was used for statistical analysis and differences were considered statistically significant with a *P* value of less than 0.05.

#### Taqman PCR and Gene Expression

RNA was isolated using RNAeasy mini kit [Qiagen (#74134)], and converted to cDNA using iScript cDNA synthesis kit [BioRad (#1708891)]. The Taqman primers used for this study were purchased from Applied Biosystems (Smad3 Hs00969210_m1, Smad2 Hs00183425_m1, Smad7 Hs00998193_m1, and SGK1 Hs00985033_g1 Taqman primers). The values are represented as the difference in Ct values normalized to GAPDH for each sample.

#### Detection of Phosphorylated Smad Proteins

Peripheral blood mononuclear cells were isolated from healthy volunteers and naïve and memory CD4^+^ T cells were sorted as described previously. Cells were stimulated for 1 h with plate bound anti-CD3 and soluble anti-CD28. Thereafter, cells were washed extensively with ice cold 1× PBS and stained with live dead stain (Thermo fisher) for exclusion of any dead cell population during analysis. Cells were fixed with 2% paraformaldehyde at 37°C for 20 min in order to cross link the phospho proteins. Cells were again washed twice with ice cold 1× PBS and permeabilized with 90% ice cold methanol at 4°C for 60 min. Cells were washed again and incubated with FACS buffer (PBS + 2% FCS) to prevent non-specific binding. Cells were stained with phospho Smad specific antibodies at 4°C for 40′ according to the manufacture’s recommendations. Finally, cells were stained with appropriate fluorescent-conjugated secondary antibodies, data were acquired by BD FACSVerse and analyzed by FlowJo. Following antibodies (phosphorylation sites included in parentheses) from Abcam were used: ab53100 (pSMAD2, pS467) and ab52903 (pSMAD3, pS423 + S425) for these experiments.

## Results

### Dietary Sodium and Potassium and Risk of UC and CD

Through 2011, we documented 273 cases of CD and 335 cases of UC among 194,711 women who contributed 3,220,247 person-years of follow-up. Compared to women in the lower quintiles of dietary potassium intake, women in the highest quintile were slightly older, more likely to live in a southern latitude, had a higher intake of daily fiber, and less likely to have never smoked (Table 1). There were no significant differences according to BMI, history of appendectomy, use of NSAIDs, oral contraceptives, or menopausal hormone therapy, and dietary sodium intake according to quintiles of potassium intake.

**Table 1:** **Baseline characteristics of participants according to quintiles of total dietary potassium intake**.^a^

	Q1 (*N* = 34,648)	Q2 (*N* = 34,818)	Q3 (*N* = 34,809)	Q4 (*N* = 34,835)	Q5 (*N* = 34,812)
Age (years), mean (SD)	42 (9)	42 (9)	43 (10)	44 (10)	45 (10)
Body mass index (kg/m^2^), mean (SD)	25 (6)	25 (5)	25 (5)	25 (5)	25 (5)
Smoking					
Never	60	58	56	54	50
Past	21	25	27	29	31
Current	19	17	17	17	19
Latitude of residence, %					
Southern latitude	14	14	14	15	16
Appendectomy, %	18	19	20	20	21
Premenopause, %	73	72	69	67	64
Menopausal hormone therapy, %^b^					
Never	54	53	53	54	53
Past	20	21	21	21	21
Current	26	26	26	25	26
Ever use of oral contraceptives, %	70	69	68	67	65
Regular use of NSAIDs, %	12	12	11	12	13
Fiber intake (g/day), mean (SD)	14 (3)	16 (4)	17 (4)	19 (5)	22 (6)
Potassium intake (mg/day), mean (SD)	2,218 (655)	2,701 (703)	2,996 (754)	3,279 (824)	3,754 (1,059)
Sodium intake (mg/day), mean (SD)	1,916 (691)	2,055 (696)	2,091 (689)	2,098 (694)	2,079 (748)

*^a^Values are means (SD) or percentages and are standardized to the age distribution of the study population. All variables are derived from baseline questionnaires (1984 in NHS and 1991 in NHSII) with the exception of geographic location (1992 in NHS and 1993 in NHSII) and appendectomy (baseline in NHS and 1995 in NHSII)*.

*^b^Percentages among postmenopausal women*.

Compared to women in the lowest quintile of dietary potassium intake, the multivariable-adjusted HRs for CD were 0.91 (95% CI, 0.64–1.29) for women in the second quintile, 0.77 (95% CI, 0.53–1.11) for the third quintile, 0.58 (95% CI, 0.38–0.88) for the fourth quintile, and 0.62 (95% CI, 0.40–0.95) for the highest quintile (*P*_trend_ = 0.005) (Table 2). Similarly, dietary potassium was inversely associated with risk of UC, although the association did not reach statistical significance (*P*_trend_ = 0.08). We did not find an association between dietary sodium and risk of CD (*P*_trend_ = 0.44) or UC (*P*_trend_ = 0.77) (Table 2).

**Table 2:** **Dietary sodium and potassium intake and risk of Crohn’s disease and ulcerative colitis**.

	Q1	Q2	Q3	Q4	Q5	*P*_trend_^b^
**Dietary potassium intake**						
**Crohn’s disease**						
Cases/person-years	68/637,956	65/646,862	55/647,848	41/647,065	44/640,516	
Age-adjusted HR, 95% CI	1.00	0.94 (0.67–1.33)	0.79 (0.55–1.13)	0.59 (0.40–0.88)	0.63 (0.43–0.92)	0.002
MV-adjusted HR, 95% CI^a^	1.00	0.91 (0.64–1.29)	0.76 (0.52–1.11)	0.58 (0.38–0.88)	0.62 (0.40–0.95)	0.005
**Ulcerative colitis**						
Cases/person-years	71/637,956	74/646,862	71/647,848	61/647,065	58/640,516	
Age-adjusted HR, 95% CI	1.00	1.02 (0.74–1.42)	0.97 (0.69–1.34)	0.83 (0.59–1.17)	0.80 (0.56–1.13)	0.09
MV-adjusted HR, 95% CI^a^	1.00	1.00 (0.71–1.39)	0.94 (0.66–1.33)	0.79 (0.54–1.15)	0.74 (0.50–1.11)	0.08
**Dietary sodium intake**						
**Crohn’s disease**						
Cases/person-years	53/638,430	54/645,396	54/645,264	37/646,930	75/644,226	
Age-adjusted HR, 95% CI	1.00	1.01 (0.69–1.48)	1.01 (0.69–1.47)	0.69 (0.45–1.06)	1.41 (0.99–2.01)	0.25
MV-adjusted HR, 95% CI^a^	1.00	1.00 (0.68–1.46)	0.98 (0.67–1.44)	0.67 (0.44–1.02)	1.32 (0.92–1.89)	0.44
**Ulcerative colitis**						
Cases/person-years	62/638,430	68/645,396	66/645,264	72/646,930	67/644,226	
Age-adjusted HR, 95% CI	1.00	1.10 (0.78–1.56)	1.05 (0.74–1.48)	1.15 (0.82–1.61)	1.07 (0.75–1.51)	0.68
MV-adjusted HR, 95% CI^a^	1.00	1.08 (0.77–1.53)	1.03 (0.73–1.46)	1.12 (0.80–1.58)	1.04 (0.73–1.48)	0.77

*CI, confidence interval; Q, quintiles*.

*^a^Models adjusted for age (months), smoking (never, past, current), body mass index at baseline (<20, 20–24.9, 25–29.9, ≥30 kg/m^2^), oral contraceptive use (ever, never), hormone therapy (never, past, current, premenopause), appendectomy (no, yes), geographic latitude of residence at age 30 (southern, middle, northern, missing/unknown), updated physical activity (quintiles), cohorts (NHS, NHSII), NSAID’s use (<2 tablets/week, ≥2 tablets/week), updated fiber intake (quintiles), and total caloric intake*.

*^b^P_trend_ was calculated by entering the median value for each quintile into the model as a continuous variable*.

We explored the possibility that pre-diagnosis symptoms may have altered dietary intake and therefore could account for our observed inverse association between dietary potassium and risk of CD. Thus, we performed sensitivity analysis using dietary data derived at least 4 years prior to each 2-year follow-up cycle and observed similar associations. Compared to women in the lowest quintile of dietary potassium, the multivariable-adjusted HR of CD among women in the highest quintile of dietary potassium was 0.60 (95% CI, 0.37–0.95) (*P*_trend_ = 0.002). Similarly, we did not find an association between dietary potassium and risk of UC (*P*_trend_ = 0.54). Compared to women in the lowest quintile of dietary potassium, the MV-adjusted HR of UC among women in the highest quintile was 1.00 (95% CI, 0.65–1.53).

We examined the possibility that specific food items with the highest contribution to dietary potassium and not cumulative daily potassium intake may explain our observed association and therefore examined the independent effect of skim milk (~9% of daily potassium), potato (~6% of daily potassium), and orange juice (~3% daily potassium) on risk of CD and UC. We did not observe any inverse association between orange juice, skim milk, and potato and risk of CD (all *P*_trend_ > 0.20). Similarly, we did not observe an association between food items with the highest contribution to dietary potassium and risk of UC (all *P*_trend_ > 0.50). In addition, we assessed the independent effect of supplements containing potassium such as multivitamins on risk of CD and UC and observed no associations (all *P*_trend_ > 0.30).

As food items with high fiber content, particularly cruciferous vegetables, also contain large amounts of potassium, we hypothesized that previous inverse associations reported between dietary fiber intake and risk of CD may be mediated by potassium (29). In a mediation analysis, we estimate that, dietary potassium intake appeared to contribute to nearly 90% (95% CI, −32 to 210%) of the association of fiber intake on CD. Specifically, the risk of CD with each one-category increase in quintile category of fiber intake was 0.92 (95% CI, 0.85–1.00) before adjustment for potassium (hypothesized intermediate) and 0.99 (95% CI, 0.89–1.10) after adjustment. However, a formal test of mediation did not reach statistical significance (*P* = 0.15).

### Interaction between Dietary Potassium and SNPs in T_H_17 Pathway

We explored the possibility of gene–environment interaction between dietary potassium and the T_H_17 pathway on risk of CD and UC in our nested case–control study (Table 3). Similar to our primary analyses, dietary potassium appears to to be inversely associated with risk of CD but not UC. Specifically, each 200 mg increase in dietary of potassium was associated with a 7% reduction in risk of CD (OR = 0.93, 95% CI, 0.86–1.00). Although we did not observe a significant interaction between dietary potassium and specific SNPs in *STAT3, JAK2, CCR6*, and *IL10* (all *P*_interaction_ > 0.15), the association of dietary potassium with risk of CD appeared to be modified by rs7657746 (*IL21*) (*P*_interaction_ = 0.004). Among participants with GG genotype, each 200 mg increase in dietary potassium was associated with an increased risk of CD (OR = 1.58, 95% CI, 1.15–2.16). In contrast, each 200 mg increase in dietary potassium was associated with an OR for CD of 0.96 (95% CI, 0.86–1.07) among individuals with GA genotypes and 0.90 (95% CI, 0.82–0.98) among individuals with AA genotypes. We also constructed a genetic risk score comprised of the number of CD or UC susceptibility alleles associated with the T_H_17 pathway on risk of CD. An interaction between dietary potassium and this genetic risk score approached statistical significance (*P*_interaction_ = 0.06). In addition, we observed a similar interaction between dietary potassium and rs7657746 (*IL21*) on risk of UC (*P*_interaction_ = 0.01). Particularly, similar to CD, among participants with CC genotype, each 200 mg increase in dietary potassium was associated with an OR for UC of 0.90 (0.83–0.98).

**Table 3:** **Risk of Crohn’s disease and ulcerative colitis according to dietary potassium intake in strata of SNPs in T_H_17 Pathways**.^a^

		Control (*n* = 740)	Crohn’s disease (*n* = 169)	Ulcerative colitis (*n* = 202)
Entire cohort (NHS + NHSII)		1.00	0.93 (0.86–1.00)	0.98 (0.91–1.05)
rs7657746 (*IL21*)	GG (*n* = 77)	1.00	1.58 (1.15–2.16)	1.12 (0.90–1.54)
	AG (*n* = 382)	1.00	0.96 (0.86–1.07)	1.02 (0.93–1.12)
	AA (*n* = 613)	1.00	0.90 (0.82–0.98)	0.90 (0.83–0.98)
	*P*_interaction_^b^		**0.004**	**0.01**
rs10758669 (*JAK2*)	AA (*n* = 491)	1.00	0.89 (0.81–0.98)	0.97 (0.89–1.06)
	AC (*n* = 393)	1.00	0.98 (0.88–1.10)	0.97 (0.87–1.07)
	CC (*n* = 177)	1.00	1.02 (0.86–1.21)	1.04 (0.90–1.19)
	*P*_interaction_^b^		0.12	0.58
rs12942547 (*STAT3*)	GG (*n* = 174)	1.00	0.93 (0.78–1.11)	0.97 (0.84–1.12)
	AG (*n* = 393)	1.00	0.97 (0.87–1.08)	1.03 (0.93–1.14)
	AA (*n* = 421)	1.00	0.92 (0.82–1.02)	0.93 (0.85–1.03)
	*P*_interaction_^b^		0.48	0.42
rs1819333 (*CCR6*)	GG (*n* = 246)	1.00	0.93 (0.80–1.07)	0.96 (0.86–1.08)
	GT (*n* = 486)	1.00	0.91 (0.82–1.00)	0.93 (0.85–1.02)
	TT (*n* = 364)	1.00	1.01 (0.91–1.12)	1.02 (0.92–1.13)
	*P*_interaction_^b^		0.18	0.41
rs3024505 (*IL10*)	GG (*n* = 773)	1.00	0.93 (0.86–1.00)	0.96 (0.89–1.03)
	AG (*n* = 276)	1.00	1.03 (0.89–1.19)	1.06 (0.94–1.19)
	AA (*n* = 30)	1.00	0.68 (0.34–1.36)	0.83 (0.62–1.12)
	*P*_interaction_^b^		0.73	0.92

*Bold values representing statistically significant P-value*.

*^a^Odds ratios are calculated for every 200 mg increase in dietary potassium intake*.

*^b^Models were adjusted for same variables as in Table 2 plus ethnicity*.

### Effect of Extracellular Potassium on Generation of Foxp3^+^ Treg Cells

**Figure 1:**
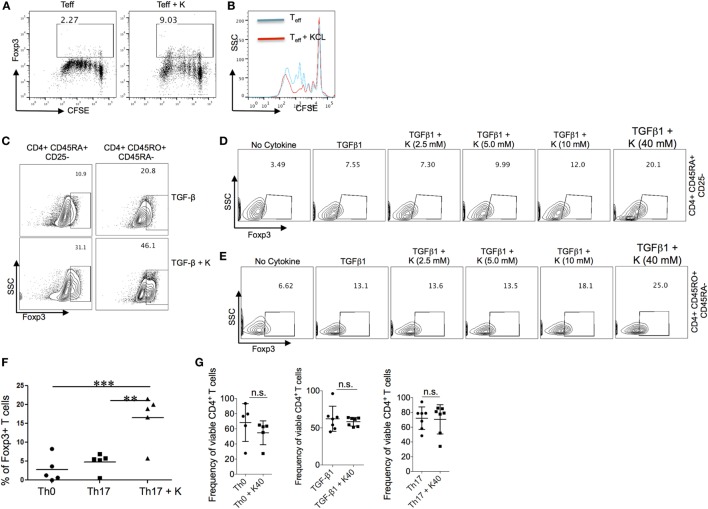
**Effect of extracellular potassium on generation of Foxp3^+^ Treg cells**. **(A,B)** Naïve (CD4^+^ CD45RO^−^CD25^−^) and memory (CD4^+^CD45RO^+^) CD4^+^ T cells were sorted and stimulated *in vitro* with anti-CD3 and anti-CD28 in presence of KCl (40mM) and CFSE, and Foxp3 expression was determined by intracellular staining and analyzed by flow cytometry after 6 days *in vitro*. **(C)** Naïve (CD4^+^ CD45RO^−^CD25^−^) and memory (CD4^+^CD45RO^+^) CD4^+^ T cells were sorted and stimulated *in vitro* with anti-CD3 and anti-CD28 in presence of TGF-β (2 ng/ml) with and without KCl (40mM), Foxp3 expression was determined by intracellular staining and analyzed by flow cytometry after 6 days *in vitro*. **(D,E)** Naïve (CD4^+^ CD45RO^−^CD25^−^) and memory (CD4^+^CD45RO^+^) CD4^+^ T cells were sorted and were stimulated *in vitro* with anti-CD3 and anti-CD28 in presence of TGF-β (2 ng/ml) and indicated doses of KCl, after 5–6 days, culture cells were analyzed for the intracellular levels of Foxp3 protein by flow cytometry. **(F)** Sorted naïve human CD4^+^ T cells were activated with anti-CD3 and anti-CD28, cells were polarized into T_H_17 conditions (TGF-β1 + IL-6 + IL-21 + IL-23 + IL-1β) *in vitro* either in normal media or media containing potassium (40mM), polarized T_H_17 cells were analyzed by flow cytometry after 6 days for percentage of Foxp3-expressing CD4^+^ T cells. Data are a cumulative plot of five individuals, each dot represents one individual (*n* = 5). Error bars SD **P < 0.01, ***P < 0.001 (Student’s *t*-test). **(G)** Naïve CD4^+^ T cells were activated with anti-CD3 and anti-CD28, cells were either polarized into T_H_17 culture condition (TGF-β1 + IL-6 + IL-21 + IL-23 + IL-1β) or TGF-β1 alone or without any cytokines (T_H_0) either in normal media or media containing potassium (40mM), cells were analyzed by flow cytometry for live/dead stain (Life technologies) by cell viability assay and represented as frequency of viable CD4^+^ T cells. Data are a cumulative plot of six to seven individuals. Each dot represents one individual. n.s., non-significant.

Immune activation and tolerance in autoimmune diseases are mediated by the interactions between genes and environment. Experimental data suggest that higher physiologic salt concentration enhances the induction of proinflammatory T_H_17 cells in mice and humans (5–7). More recently, it has also been demonstrated that high salt diet inhibits the suppressive functions of Treg cells (8). Based on our findings in the NHS cohorts and in light of animal and human studies that have supported a role for sodium on regulation of immune tolerance and development of autoimmunity, we hypothesized that potassium, in contrast to sodium, may have an opposing effect on regulatory and effector T cells. To test our hypothesis, we activated the naïve CD4^+^ T cells in the presence of potassium and tested their proliferation. Potassium concentrations were selected based on its known physiologic concentrations in the gut lumen, mucosal cells, and extracellular fluids (37). As expected, potassium suppressed the T cells proliferation and induced Foxp3 expression (Figures 1A,B). Since TGF-β1 induces the generation of Foxp3^+^ Tregs cells, next we tested whether potassium can further enhance the induction of TGF-β-induced Foxp3^+^ Tregs cells. To do this, we activated the sorted naïve human T cells with TGF-β1 in the presence and absence of potassium. As previously suggested, TGF-β1 treatment induced the expression of Foxp3 (~11%). Moreover, the addition of potassium further enhanced the generation of TGF-β-induced Foxp3^+^ Treg cells (from ~11% to ~31%) (Figure 1C). We further tested whether the enhancing effect of potassium on induction of Foxp3 expression is restricted to naïve CD4^+^ T cells. Addition of potassium also enhanced the generation of TGF-β1-induced Foxp3^+^ Treg cells (~ 21% to ~ 46%) (Figure 1C). Potassium at various concentrations also enhanced Foxp3 expression in sorted naïve and memory cells derived from healthy individuals cultured with TGF-β1 (Figures 1D,E). With 10mM of KCl, the expression of Foxp3 was nearly doubled (~7% to ~12%), and further enhanced to nearly threefold with 40 mM KCl (from ~7% to ~20%) (Figure 1E). We observed a similar effect on TGF-β1-induced Foxp3 expression on sorted memory CD4^+^ T cells.

TGF-β1 is a common differentiation factor for the generation of T_H_17 and Foxp3^+^-induced regulatory T cells (iTregs) cells. Proinflammatory cytokines, IL-6, IL-1β, IL-21, and IL-23 suppress the generation of TGF-β1-induced Treg cells by inhibiting the expression of Foxp3, which in turn favor development of T_H_17 cells. Since potassium enhanced the generation of Foxp3^+^ Tregs induced by TGF-β1, we tested whether potassium can suppress the inhibitory effect of T_H_17 cells inducing cytokines such as IL-6 and IL-1β, and therefore reinforce the expression of Foxp3 in T_H_17 cells. The addition of potassium in T_H_17 culture condition overcame the inhibitory effect of IL-6/IL-1β on Foxp3^+^ Tregs and reinforced the expression of Foxp3 in T_H_17 cells (Figure 1F). We also evaluated whether high doses of potassium in these assays may induce T cells death. Sorted T cells were cultured in the presence or absence of 40 mM potassium and tested their viability. We did not find any difference in the viability of T cells cultured with or without potassium (Figure 1G). Taken together these results demonstrate that potassium, in contrast to sodium, enhances the generation of Foxp3^+^ Treg cells without affecting the cell viability.

### Mechanism of Potassium-Mediated Generation of Foxp3^+^ Tregs

We explored the potential mechanisms by which potassium enhanced expression of Foxp3 by evaluating its effect on Smad pathway. It is known that Smad2/3 pathway is critically essential for TGF-β1-mediated induction of Foxp3 while inhibition of Smad7 is crucial for generating and maintaining Treg phenotype (38–40). *Smad2/Smad3* double deficient mice develop fatal inflammatory diseases with reduction in Foxp3 expression in CD4^+^ T cells while *Smad2* deficiency make mice susceptible to DSS induced colitis (39). In addition, Smad7 appears to be over-expressed in purified mucosal T cells from CD patients and its inhibition restores TGF-β1 signaling by enhancing the functions of Smad3 (40). Interestingly, oral SMAD7 antisense oligonucleotide was recently successfully used for treatment of CD in randomized clinical trial (41).

**Figure 2:**
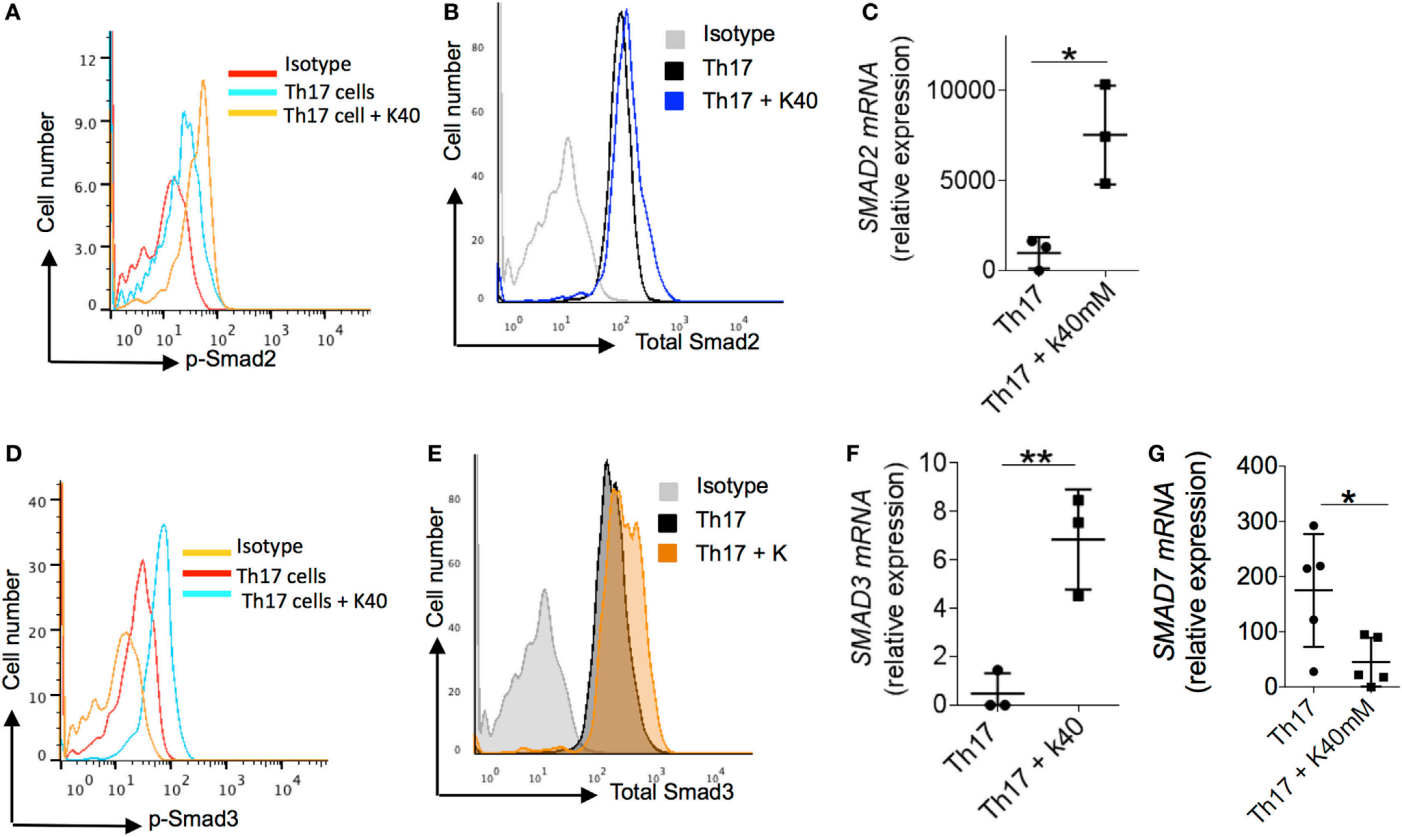
**Mechanism of extracellular potassium-mediated generation of Treg**. **(A–C)** Naïve (CD4^+^ CD45RO^−^CD25^−^) CD4^+^ T cells were sorted from total PBMCs, cells were stimulated *in vitro* with anti-CD3 and anti-CD28 and polarized into T_H_17 cells with and without potassium (40mM). After 24 h of *in vitro* polarization, cells were analyzed by flow cytometry for the phosphorylated levels of Smad2 **(B)** and total levels of Smad2 protein **(C)**. Cells were analyzed at 48 h for the expression of *Smad2* mRNA by q-RT PCR **(D)**, data are a cumulative representative plot of three individuals (*n* = 3), each dot represents one individual. *P < 0.05 (Student’s *t*-test). **(E,F)** Naïve (CD4^+^ CD45RO^−^CD25^−^) CD4^+^ T cells were sorted from total PBMCs. Cells were stimulated *in vitro* with anti-CD3 and anti-CD28 and polarized into T_H_17 cells with or without potassium (40mM). After 24 h of *in vitro* polarization, cells were analyzed by flow cytometry for the phosphorylation of Smad3 **(E)** and total levels of Smad2 protein **(F)** and mRNA expression was analyzed at 48 h for the Smad3 by q-RT PCR, data are a cumulative representative plot of three individuals (*n* = 3), each dot represents one individual, **P < 0.01 (Student’s *t*-test). **(G)** Naïve (CD4^+^ CD45RO^−^CD25^−^) CD4^+^ T cells were sorted from total PBMCs and *in vitro* stimulated with anti-CD3 and anti-CD28 with T_H_17 culture condition with or without potassium (40mM). After 48 h of activation, cells were analyzed by q-RT PCR of *Smad7* mRNA. Representative plot of five individuals (*n* = 5), each dot represents one individual, *P < 0.05 (Student’s *t*-test).

Since potassium enhanced the function of TGF-β1 in inducing the expression of Foxp3, we tested the effect of potassium on Smad2, Smad3, and Smad7 in human T cells by measuring its phosphorylation in T_H_17 cells. We found that potassium increased phosphorylation of Smad2 in T_H_17 cells (Figure 2A) and enhanced expression of total Smad2 in T_H_17 cells (Figure 2B). To further confirm this observation, we also tested the mRNA expression of *Smad2* in T_H_17 cells. Potassium significantly increased the expression of *Smad2* in T_H_17 cells (Figure 2C) and phosphorylation and expression of Smad3 (Figures 2D–F). Since the absence of Smad7 is required for the positive auto-regulatory loop of TGF-β1 on regulatory T cells, we explored whether potassium also suppresses the expression of *Smad7* in T_H_17 cells. Potassium significantly suppressed the expression of Smad7 in T_H_17 cells (Figure 2G), which may in turn reinforce the expression of Foxp3 and Treg cells phenotypes. Taken together, these observations imply that exposure of potassium to T cells increased the generation of Foxp3 by activating Smad2/3 and inhibiting Smad7 expression.

## Discussion

In two large prospective cohorts of U.S. women, we show that dietary potassium, but not sodium, is inversely associated with risk of CD. Notably, we observed a significant interaction between dietary potassium and rs7657746 (*IL21*) in the T_H_17 pathway, supporting a biological mechanism that may mediate the effect of potassium on development of CD. IL-21 plays a key role in development of T_H_17 cells through STAT3, a transcription factor required for the differentiation of T_H_17 cells *in vivo* (42). IL-21 and IL-23 induce the orphan nuclear receptor RORγ, which in synergy with STAT3 promotes IL-17 expression in CD4^+^ T cells, leading to induction of T_H_17 cells (42). In addition, IL-21 inhibits the generation of FoxP3^+^ Treg induced by TGF-β to induce T_H_17 cells. We corroborated our findings by showing *in vitro* that potassium regulates Treg/T_H_17 balance through induction of Foxp3 expression in naïve and memory T cells as well as T_H_17 cells even in the presence of proinflammatory cytokines. This effect appeared to be mediated through increase in both phosphorylation and expression of Smad2 and Smad3 and suppression of Smad7 expression.

In contrast to potassium, recent data have shown a proinflammatory role for sodium mediated by the induction and generation of pathogenic T_H_17 cells in human and mice (5, 6). Moreover, a high sodium diet enhances the susceptibility of tissue inflammation in experimental autoimmune encephalomyelitis in animal models with augmented T_H_17 cell response (5). Interestingly, the proinflammatory effect of sodium appears to be further enhanced through marked reduction in Foxp3^+^ Tregs functions (8). T_H_17 cells and Tregs can be induced reciprocally, as TGF-β alone generates Tregs by inducing the expression of Foxp3 while addition of IL-6 together with TGF-β suppresses the expression of Foxp3 and generates IL-17-producing T_H_17 cells (43). In contrast to the effect of sodium on T_H_17 cells and Tregs, our findings show that potassium not only enhances the induction of Foxp3 induced by TGF-β1 but also reinforces Foxp3 expression in T_H_17 cells suggesting a possible anti-inflammatory function of potassium *via* induction of Foxp3-mediated T cells tolerance.

Consistent with our observation that potassium may have anti-inflammatory effects on activated T_H_17 cells, Wen and colleagues recently demonstrated that potassium supplementation in healthy volunteers blocks IL-17 production by T cells previously treated with sodium. Interestingly, the effect appears to be mediated through the direct suppression of p38/MAPK-SGK1 pathway (44). As SGK1 has a critical role in the induction of pathogenic T_H_17 cells in response to modest increase in sodium concentration (6), it appears that the balance between T_H_17 and Treg cells may in part be regulated by concentration of sodium and potassium. In support of this hypothesis, we also demonstrate that potassium supplementation, in contrast to sodium, inhibits the expression of *SGK1* (Figure S1 in Supplementary Material). Similar to our findings, a recent study by Eil and colleagues also demonstrated the profound suppressive effect of potassium, released into the extracellular fluid as a result of necrosis in human tumors, on T cell effector function (45).

The plasticity of T_H_17 and Treg cells is well established as human Tregs, in a given proinflammatory environment can adopt T_H_17 phenotype and express IL-17 (46–48). Similarly, Foxp3-expressing human T_H_17 cells retain suppressive functions (47). Our data therefore suggest that potassium induces the T_H_17-Tregs plasticity by inducing Foxp3 in T_H_17 cells. Whether potassium-mediated induction of Foxp3 into T_H_17 cells leads these cells to develop a suppressive phenotype is the topic of future work. Lastly, IL-21 appears to play a key role in development of T_H_17 cells through STAT3, a transcription factor required for the differentiation of T_H_17 cells *in vivo* (42). However, it is not clear whether our gene–environment interaction data suggest a specific role for IL-21 in potassium-mediated induction of Foxp3.

Our study has several notable strengths, including prospectively collected and updated information on diet and other important life style factors, corroborative findings from gene–environment interactions, and compelling correlative *in vitro* functional studies. Taken together, these provide complementary lines of evidence that enhance the likelihood that the association between dietary potassium and IBD is causal. We acknowledge limitations of our study, including measurement errors arising from use of FFQs to estimate dietary potassium and sodium and a limited sample size in our analyses of gene–environment interaction. Lastly, we acknowledge that potassium concentration is tightly regulated in the extracellular fluids (~5 mEq/L). However, its concentration in the gut lumen and the mucosal cells vary significantly (16–130 mEg/L), therefore, concentrations (2.5–40 mM) used in our *in vitro* experiments appear to be in physiologic range. In addition, similar concentrations were used in recent study evaluating the inhibitory effect of potassium on antitumor T cell response (45).

In conclusion, we show that dietary potassium is inversely associated with risk of CD with both *in vitro* and gene–environment interaction data suggesting a potential role for potassium in regulating immune tolerance through its effect on Tregs and T_H_17 pathway. To our knowledge this is the first study demonstrating a potential interaction between dietary intake, genetic risk, and immune function on risk of developing CD. Further research to better elucidate the precise mechanisms by which dietary potassium may modulate immune tolerance through regulating the T_H_17/Tregs balance is warranted. Finally, investigating a role for dietary potassium in modulating disease activity among patients with established CD and specific genotype should be a high priority.

## Ethics Statement

This study was carried out in accordance with the recommendations of “Human Research Committee at Partners Healthcare” with written informed consent from all subjects. All subjects gave written informed consent in accordance with the Declaration of Helsinki. The protocol was approved by the “Human Research Committee at Partners Healthcare.”

## Data Sharing

Requests for access to data, statistical code, questionnaires, and technical processes may be made by contacting the corresponding author at hkhalili@mgh.harvard.edu.

## Author Contributions

HK – study concept and design; statistical analysis; interpretation of data; and drafting of the manuscript. SM – experimental design and interpretation of data. ANA – acquisition of data and critical revision of the manuscript. JG – acquisition of data and critical revision of the manuscript. LH – acquisition of data and critical revision of the manuscript. AJ – statistical analysis; interpretation of data; and critical revision of the manuscript. JP – acquisition of data and critical revision of the manuscript. JR – acquisition of data and critical revision of the manuscript. KS – statistical analysis; interpretation of data; and critical revision of the manuscript. GC – acquisition of data; interpretation of data; and critical revision of the manuscript. AA – study concept and design; acquisition of data; drafting of the manuscript; and critical revision of the manuscript. VY – study concept and design and critical revision of the manuscript. AC – study concept and design and critical revision of the manuscript.

## Conflict of Interest Statement

Dr. AA is a member of the scientific advisory board for Exact Sciences, Abbvie, and Cubist Pharmaceuticals. Dr. JR is a consultant for Policy Analysis, Inc. Dr. AC has served as a consultant for Bayer Healthcare, Pfizer Inc., and Aralez Pharmaceuticals. Other authors have no financial disclosures. Dr. GC is a consultant for Allena Pharmaceuticals and Astra Zeneca. The other authors declare no conflict of interest.

## Supplementary Material

Figure S1 **Naïve (CD4^+^CD45RA^+^CD45RO^−^) CD4^+^ T cells were sorted from total PBMCs, cells were stimulated *in vitro* with anti-CD3 and anti-CD28 and polarized into T_H_17 cells with and without potassium as indicated, cells were analyzed at 48 h for the expression of *SGK1* mRNA by q-RT PCR, data are a cumulative representative plot of three individuals (*n* = 3)**. Each dot represents one individual. **P* < 0.05 (Student’s *t*-test).
