# Supplementary figures and images for: Identification and Characterization of a Novel Association between Dietary Potassium and Risk of Crohn’s Disease and Ulcerative Colitis
Source: Front Immunol. 2016 Dec 7;7:554. doi: 10.3389/fimmu.2016.00554 (PMC5141241; doi:10.3389/fimmu.2016.00554)

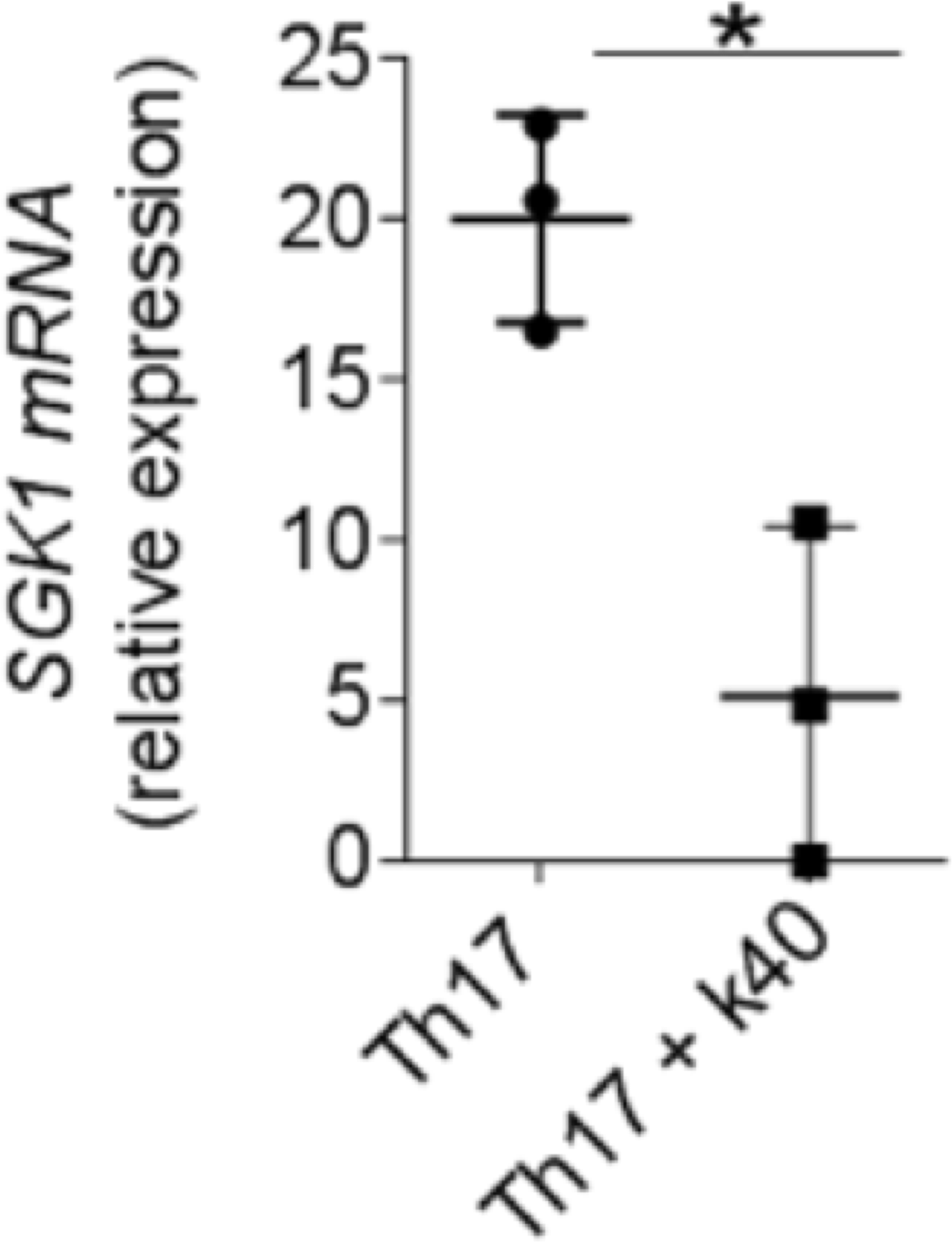

Supplement: Figure S1 — Naïve (CD4+CD45RA+CD45RO−) CD4+ T cells were sorted from total PBMCs, cells were stimulated in vitro with anti-CD3 and anti-CD28 and polarized into TH17 cells with and without potassium as indicated, cells were analyzed at 48 h for the expression of SGK1 mRNA by q-RT PCR, data are a cumulative representative plot of three individuals (n = 3). Each dot represents one individual. *P < 0.05 (Student’s t-test). [file image_1.tiff]
